# Sleep Bruxism and SDB in Albanian Growing Subjects: A Cross-Sectional Study

**DOI:** 10.3390/dj9030025

**Published:** 2021-02-27

**Authors:** Giuseppina Laganà, Vesna Osmanagiq, Arianna Malara, Nicolò Venza, Paola Cozza

**Affiliations:** 1 Department of Systems Medicine, University of Rome ‘Tor Vergata’, Via Montpellier 1, 00133 Rome, Italy; drariannamalara@gmail.com (A.M.); venza.nicolo@gmail.com (N.V.); profpaolacozza@gmail.com (P.C.); 2 Department of Dentistry UNSBC, 1026 Tirana, Albania; v.osmanagiq@fzkm.org

**Keywords:** bruxism, questionnaire, SDB, OSAS

## Abstract

The aim of this study was to evaluate a possible correlation between sleep bruxism and risk factors of developing obstructive sleep apnea syndrome (OSAS) in a sample of growing subjects and to assess parental awareness about sleep bruxism in their children. **Methods:** The sample was composed of 310 subjects (173 females and 137 males), with a mean age of 8.9 years, attending “Ndre Mjeda” school of Tirana (Albania). All parents of the children participating in the study were asked to fill in a questionnaire manually or via a digital version. The questionnaire was composed of three sections: personal data, sleep quality data, and OSAS risk factors, and it was filled out by both parents. **Results:** Of our samples, 41.3% presented with bruxism, and 16.5% of the parents ground their teeth. Oral breathing was reported in 11.9% of the subjects, and among these, 40% of the subjects were affected by bruxism (*p* > 0.05). Of the subjects, 18.7% snore overnight. Comparing it with sleep bruxism, the two phenomena are often related (*p* = 0.00). **Conclusions**: Heredity, night-sweating, nycturia, oral breathing, and snoring seem to have a significant correlation with bruxism.

## 1. Introduction

Sleep bruxism is a masticatory muscle activity during sleep that is characterized as rhythmic (phasic) or non-rhythmic (tonic), and is not a movement disorder or a sleep disorder in otherwise healthy individuals [1]. In these cases, bruxism should be considered as a behavior that can be a risk factor for certain clinical consequences [1]. As a matter of fact, it is a phenomenon which is gaining increasing attention in the dental and medical literature for its relationship with several disorders, such as dental problems, orofacial pain, neurological diseases, and breathing disorders [2].

A wide variety of cases is present in the literature reporting the prevalence of bruxism (between 6–91%) with no gender difference [3], although some studies underline a higher prevalence of female cases [4], and a decrease in prevalence with age [4] from children (2–30%) to adults (5–10%), and especially in the elderly population (2–4%) [5]. This variability is mainly due to the study method of the prevalence of sleep bruxism in children. Among sleep disorders recognized in children, sleep bruxism is one of the most commonly reported [5]. Although there is less supporting literature, awake bruxism has been reported to have a prevalence of around 22% [6].

Approaches for assessing bruxism can be distinguished as non-instrumental or instrumental. Non-instrumental approaches include self-report (questionnaires, oral and dental history) and clinical inspection, both for sleep and awake bruxism [7]. However, tooth-based diagnostic tools are often used, and this is not a reliable measure, because of the nonlinear relationship between bruxism and tooth wear [8].

Instrumental approaches are currently available for both sleep and awake bruxism and use electromyographic recordings often associated with other measures used in somnography or polysomnography (e.g., audio and/or video recordings) [9].

Due to the lack of a valid and large-scale diagnostic tool, most of the epidemiological data derive from non-instrumental approaches, like questionnaires by the patients and the bed partners.

Sleep-Disordered Breathing (SDB) is characterized by abnormal respiration during sleep. These disorders are divided into obstructive sleep apnea (OSA), central sleep apnea, sleep-related hypoventilation, and sleep-related hypoxemia disorder [10].

SDB are commonly reported in 25–40% of preschoolers and school children; many conditions ranging from primary snoring to obstructive sleep apnea syndrome (OSAS) are included [11]. OSAS is a serious public health problem due to its frequency and numerous pathophysiological consequences, such as excessive daytime sleepiness, increased risk of cardiovascular disease, and reflux esophagitis [12].

Key risk factors for OSAS include fat deposition in upper airway tissues due to obesity, adeno-tonsillar hypertrophy, nasal congestion secondary to hypertrophic rhinitis or allergic rhinitis, and tonsil hypertrophy [13]. According to recent estimations, the incidence of OSAS in children ranges from 1% to 3%, and increases to 60% in high-risk patients, such as overweight and obese children [14]. Some studies report concomitant disturbances of sleep bruxism and obstruction of sleep apnea-hypopnea [13] and a positive correlation between sleep bruxism events and obstructive apnea, suggesting that OSAS could be an important risk factor for sleep bruxism [15,16,17]. In addition, children with sleep bruxism can have a high likelihood of showing daytime problematic behavior, which can also be frequently associated with sleep problems [18,19]. Otherwise, some authors postulated that sleep bruxism might have a protective role during sleep, which may relate to airway maintenance [20] or in stimulating saliva flow to help the oropharynx [21]. Therefore, a positive relationship between these two phenomena can exist, but the strength and specificity of this association is still debated in the literature [22].

Identifying the presence of risk factors for OSAS can allow for early diagnosis, effective therapy, and prevent other physical diseases [23,24].

The present study represents the first application of assessing bruxism by a non-instrumental approach in Albania’s growing population. It aimed to evaluate, by a questionnaire, a possible correlation between sleep bruxism and the risk factors for developing OSAS in a large sample of growing subjects. Furthermore, the second aim was to assess parental awareness about sleep bruxism in children and its relations with general health. The null hypothesis of this study is that there is a correlation between sleep bruxism and SDB in the Albanian growing population.

## 2. Materials and Methods

**Figure 1:**
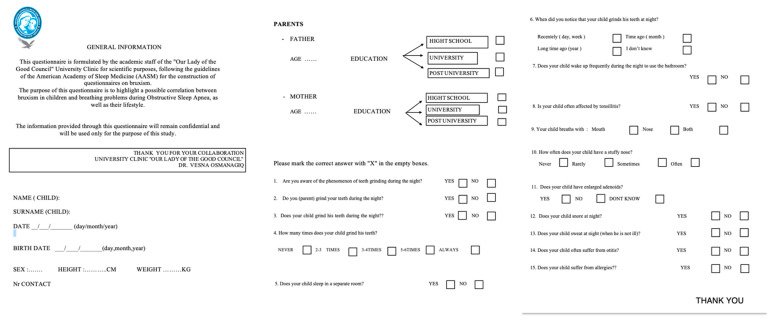
Complete English questionnaire.

This study followed the principles laid down by the World Medical Assembly in the Declaration of Helsinki 2008 on medical protocols and ethics, and it received a positive response by the Ethical Committee at the University of “Our Lady of Good Counsel” in Tirana (n°139/2019). For each study participant, written consent was obtained from both parents of the children. The study population was selected from October 2018 until June 2019, and it was composed of 310 subjects (173 females and 137 males), from 6 to 12 years old (mean age 8.9 years). The participants were attending the “Ndre Mjeda” school of Tirana (Albania). All the subjects had not undergone orthodontic treatment either before or during the compilation. Children with severe systemic diseases, secondary bruxism induced by systemic diseases and/or drugs, use of medicines that can significantly affect the function of the nervous and muscular systems, severe mental illness, or significant mental disorders were excluded. All parents of the children participating in the study were asked to fill in a questionnaire manually or via the digital version. The original questionnaire was constructed in the Albanian language and completed by both parents of the children participating in the entrance to the University Clinic “Our Lady of Good Counsel”; no significative differences were found between mothers and fathers. The completed questionnaires were delivered by the teachers at the participating schools to the parents of the children and also in Google forms online. In the questionnaire, the word “bruxism” was replaced with the word “grinding” to make the phenomenon more understandable for parents. The questionnaire was composed of three sections: 1-Personal data: physical and demographic data of the children and the demographic and educational level of the parents; 2-Sleep quality: data about the consciousness of grinding, snoring, and sleep routines during the night; 3-OSAS risk factor: adenotonsillar hypertrophy, nasal congestion, allergic rhinitis and tonsil hypertrophy, otitis, sweating, and nighttime hyperactivity. The complete questionnaire is shown in Figure 1.

### 2.1. Questionnaire Validation

The questionnaire was designed by one of the investigators (V.O.) of the study with experience in this field, while the survey content and wording were revised by the other members of the team. Interviews on the content and wording of the questionnaire were conducted on 100 parents of the children involved in the study. Both parents were interviewed by the clinician who developed the questionnaire after they had completed it. Subjects were asked whether the items adequately captured the extent of their children’s symptoms. Any problems with the design or wording of the questions and interpretation were researched. The reworded questionnaire was presented to half of the subjects recruited for review. No additional revisions or changes were needed. For the validation of the new survey, 15% of the subjects were recruited. They were randomly selected from the total sample of 100 in order to guarantee that there were no differences in age or gender of the smaller subgroup compared to the complete study population.

### 2.2. Statical Analysis

Data processing was carried out using software SPSS Statistics (Statistical Package for Social Sciences, version 20.0, SPSS Inc., Chicago, USA). A descriptive analysis of all the variables considered was initially carried out, in order to identify any anomalous or incorrect data and to provide a concise overview of the sample considered. The Chi Square test was used to highlight the link between the variables, dividing the group into those with bruxism and without bruxism. Specifically, the statistical significance of the patients who ground their teeth and the risk factors was assessed. The results were also considered with Fisher’s Exact Test. Values of *p* < 0.05 were considered significant.

## 3. Results

The sample was composed of 310 subjects (173 F, 56% of the total sample, 137 M, 44% of the total sample) between 6 and 12 years of age with a mean age of 8.9 years (SD = 2). The height of the subjects varies from 100.0 to 170.0 cm (mean 135.7 cm, SD = 14.3). The weight of the subjects is between 15.0 and 77.0 kg with (mean 32.9 kg, SD = 9.3). Table 1 shows the demographic characteristics of the subjects.

The answer to Question 3 was used to classify subjects as with bruxism (BG) or without bruxism (NBG): 128 subjects (41.3%) were affected by sleep bruxism, while 179 subjects (57.7%) were not affected by this behavior. In only three cases, the parents were unable to answer the question, and they were eliminated from the studied sample. Therefore, 41.3% of our samples presented with bruxism, including 43.7% females and 56.3% males. 

Table 2 shows the distribution of sleep bruxism in the sample.

Obesity was assessed as a risk factor in developing OSAS [13]; the BMI index was used to evaluate the presence of obesity in the sample and the relationship with the presence of sleep bruxism. Table 3 shows the distribution of obesity in the studied population.

The education level of the parents and their information on bruxism was assessed. As shown from the following table (Table 4), in 12.9% of cases, the male parent had a higher education level than the female parent, but both parents replied “YES” to the question, “Do you have information on night grinding?” in only 52.9% of cases.

The frequency of sleep bruxism was evaluated in parents who completed the questionnaire through the question, “Do you grind your teeth at night?” and the frequency of children who grind their teeth at night through the question, “Does your child grind his teeth during the night?”. The following tables (Table 5 and Table 6) show that only 16.5% of parents grind their teeth, compared to 41.3% of children. The comparison of the data shows that in 29 cases, sleep bruxism is present simultaneously in one of the parents and in the child (*p* = 0.001). 

Table 7 shows the correlation between sweating, nycturia, and sleep bruxism. Analysis of the responses shows that 14% of the subjects with bruxism suffer from nycturia. The Chi Square and Fisher’s Exact Test showed a significant statistical correlation of *p* < 0.05.

The frequency of tonsillitis was 22.3%. No statistical significance was found between the presence of tonsillitis and the presence of sleep bruxism. As shown in Table 8, oral breathing was reported in 11.9% of the subjects, and among these, 40% of the subjects were also affected by bruxism (*p* > 0.05). Nasal congestion was detected in 9.7% of the subjects, and 56% of them ground their teeth; however, the statistical analysis did not reveal a significance between the two factors. The parents of the subjects reported the presence of enlarged adenoids in 8.7% of cases, and among these, 33% suffered from sleep bruxism. Even in this case, no significance was found between the two factors.

Table 8 also shows the results on snoring, where 18.7% of subjects were found to snore overnight. Comparing it with sleep bruxism, the two phenomena are often related (*p* = 0.00). Of our sample, 20% suffered from allergic rhinitis, and 23 subjects exhibited a coexistence with sleep bruxism. As displayed in Table 8, Fisher’s Exact Test gave us a non-significant result among the analyzed variables.

## 4. Discussion

The purpose of the present study was to evaluate, by a questionnaire, a possible correlation between sleep bruxism and the risk factors of developing OSAS in a sample of Albanian growing subjects. Secondarily, the awareness of the parents about sleep bruxism and the impact of educational levels on its knowledge were investigated. 

The results showed that 41.3% of the sample was affected by bruxism, where in particular, males ground their teeth more than females (56.3% vs. 43.7%), although the literature underlines that females are the ones grinding more during the night [4].

Moreover, as reported in the literature, obesity is an important risk factor, both for sleep bruxism and OSAS in children [25]. For this reason, BMI analysis was performed on the whole sample, and the results showed that even if 41.3% of the sample presented with bruxism, the BMI value was defined as “underweight” in 63.4% of the sample, and this result is not supported by the literature.

Two specific symptoms important for the diagnosis of OSA and influencing the quality of sleep in these children were also evaluated: night sweating [26] and nycturia [27]. It was found that children with bruxism also presented with sweating at night (*p* = 0.02), and this result was observed in 41.6% of cases, while in 15.5%, an increase of nycturia was registered (*p* = 0.04). Several studies previously reported that SB can occur simultaneously with Sleep-Disordered Breathing (SDB), and could therefore mutually interact to increase their severity [28,29,30]. In particular, the existence of a correlation between sleep bruxism and OSA on adult subjects is mentioned in various articles: in a study conducted by Tsujisaka et al. in 2018 [31], 61 patients with OSA syndrome (diagnosed by polysomnography) were analyzed, and 22 presented with bruxism. In 2013, Saito [16] reported that 54.9% of the studied population ground their teeth after sleep apnea events to allow airway patency during sleep.

In 2014, Hosoya [15] conducted a study on 80 patients with OSAS diagnosed by polysomnography, and showed that 47.8% of subjects showed bruxism, stating that sleep apnea is a risk factor for bruxism. This study suggests a positive correlation of bruxism in OSAS patients—bruxism allows, through microarousal, a resumption of the respiratory event, freeing the airways and allowing the passage of air. On the contrary, another paper did not support the association between SB and SDB. As matter of fact, SB was not observed with snoring or apneic events in any of the subjects of the study sample, and masseter activity was not observed during apneic episodes [32].

In the current study, the OSAS’ risk factors, such as tonsillitis and enlarged adenoids, were evaluated in growing subjects with bruxism, as supported by the literature. Tonsillitis and enlarged adenoids have a frequency of 22.3% and 8.7%, respectively, in the studied sample: 31 subjects with bruxism often had tonsillitis, while only 9 had enlarged adenoids. This low value can be justified, as the enlarged adenoids do not have very important symptoms, so the parents are often unaware about this phenomenon. No positive correlation has been found between bruxism, tonsillitis, and enlarged adenoids (*p* > 0.05). 

About 11.9% of the parents of the children in the sample reported that their child breathed only through their mouth, and among these, 40% of subjects presented with bruxism (*p* > 0.05), while 50% of the parents reported that their child breathed through the nose and not through the mouth; in addition, 9.7% of the parents reported that these children often had a blocked nose, and 56% of them ground their teeth. However, the statistical analysis did not reveal a significance between the two factors.

In 2011, Bektas [33] stated that nasal obstruction can have an indirect preventive and therapeutic effect on temporomandibular disorders (TMD) related to sleep bruxism by causing mouth respiration. Nasal obstruction makes a sleep bruxism episode less possible, which, if present, would simultaneously make inspiration almost impossible. It is likely that the patient will enter an episode of apnea that should end with the opening of the mouth and/or awakening that would both end the sleep bruxism episode.

Since snoring is classified as the first stage of SDB in children [34], it is important to assess whether this factor also has a significant correlation with bruxism: 18.7% of subjects snore overnight. Comparing it with sleep bruxism, the two phenomena are often related (*p* = 0.00). Allergic rhinitis in the study had a weak or absent correlation with bruxism [34], as demonstrated in various articles.

Since there is a correlation between sleep bruxism and SDB in the Albanian growing population, the null hypothesis of this study is confirmed.

The education level of parents and their information on bruxism as a phenomenon was also assessed. In 12.9% of cases, the male parent had a higher education level than the female parent, but both replied “YES” to the question, “Do you have information on night grinding?” in only 52.9% of cases. This lack of information, regardless of the educational level of the parents, is also supported by Prado [35] in a study carried out in Brazil on a sample of 1325 parents, where 57.3% of them had no information about the phenomenon, and 88.9% said they would like to know more. This situation may be correlated with the actual low level of general prevention in the Albanian population, as described in other papers on growing subjects [36,37,38].

Regarding the heredity of bruxism and the direct transmission between parents and children, a positive correlation (*p* = 0.00) was found in this study, as also supported by the bibliographic review carried out by Lobbezoo in 2014 [39], according to which bruxism is partly hereditary. Specifically, a work by Wieckiewicz et al. suggested a possible genetic contribution of the variability within the serotonin receptor encoding gene (*HTR2A*) and possibly also within the dopamine (*DRD1*) receptor gene to the etiology of SB [40].

## 5. Conclusions

This paper highlighted important results about bruxism and Sleep-Disordered Breathing, and for the first time, showed data observed in an Albanian growing population on this topic. The null hypothesis is so confirmed.

Of our sample, 41.3% were found to grind their teeth during the night, and 46.5% of parents had no information about the phenomenon of bruxism. Tonsillitis seemed not to be correlated with sleep bruxism, and further clinical investigations are needed to confirm this relationship.

Heredity, night sweating, nycturia, oral breathing, and snoring seem to have a significant correlation with bruxism. This should be read as a wake-up call to the coexistence of these factors.

**Study limitations**: The risk of bruxism and Sleep-Disordered Breathing was subjectively determined using a questionnaire. Moreover, the sample can be more numerous, and it could be the aim of a second study on this topic, considering the great lack of epidemiological information in Albania.

## Figures and Tables

**Table 1:** Descriptive analysis of the studied sample.

**Sample**	**Total**	**n = 310**
	M	137 (44%)
	F	173 (56%)
Age	Min	6 y
	Max	12 y
	Mean	8.9 y
	SD	2
Height	Min	100.0 cm
	Max	170.0 cm
	Mean	135.7 cm
	SD	14.3
Weight	Min	15.0 kg
	Max	77.0 kg
	Mean	32.9 kg
	SD	9.3
BMI	Min	8 kg/m^2^
	Max	33 kg/m^2^
	Mean	17.8 kg/m^2^
	SD	3.3

**Table 2:** Distribution of sleep bruxism in the sample.

	N	%	M	F
BG	128	41.3%	43.7%	56.3%
NBG	179	57.7%	41.9%	58.1%
Missing	3	1%	/	/

**Table 3:** Distribution of obesity in the studied population.

BMI	Nutritional Status	Distribution
<18.5	Underweight	63%
18.5–24.9	Normalweight	33.2%
25.0–29.9	Pre-obesity	2.7%
30.0–34.9	Obesity Class I	1.1%
35.0–39.9	Obesity Class II	
>39.9	Obesity Class III	

**Table 4:** Education level of parents.

	High School	University	Post-University	Information on Bruxism
Mother	36.1%	45.8%	16.8%	52.9%
Father	51.6%	32.6%	12.9%	

**Table 5:** Distribution of sleep bruxism in parents and children.

	Yes	No
Do you grind your teeth at night?	16.5%	82.9%
Does your child grind his teeth during the night?	41.3%	57.7%

**Table 6:** CrossTab between sleep bruxism in parents and children. * *p* < 0.05.

		Do You Grind Your Teeth at Night?	
		Yes	No
Does your child grind his teeth during the night?	Yes	29 *	99
	No	21	157

**Table 7:** Correlation between sweating, nycturia, and sleep bruxism (* *p* < 0.05).

		Sweating			Nycturia	
		Yes	I Don’t Know	No	Yes	No
SB	Yes	59	0	69	21 *	107
	No	70	3	104	23	104

**Table 8:** Distribution of principle variables investigated and its correlation with sleep bruxism.

	Tonsillitis	Oral Breathing	Nasal Congestion	Enlarged Adenoids	Snoring	Allergic Rhinitis
%	22.3%	11.9%	9.7%	8.7%	18.7%	20%
SB	39%	40%	56%	33%	36%	11%

## Data Availability

The datasets used and analyzed during the current study are available from the corresponding author on reasonable request.
